# NIR imaging-guided carbon monoxide nanomedicine: Design strategies, stimuli-responsive release, and multimodal synergistic therapies for precision disease treatment

**DOI:** 10.1016/j.mtbio.2025.102700

**Published:** 2025-12-24

**Authors:** Yaoqiang Li, Yeneng Dai, Bo Wang, Nan Zhang, Kangyi Yan, Haoqin Chen, Hongrong Shi, Hongli Chen, Qingzhi Wang, Jierui Yan, Xiaobo Wang, Peiyang Gao, Gongcheng Ma, Ya Hou, Qihang Ding, Qi Zhao

**Affiliations:** a Xinxiang Key Laboratory of Biomedical Materials, Nanobiomedical Materials Research Center, School of Life Science and Technology, Xinxiang Medical University, Xinxiang 453003, China; b MoE Frontiers Science Center for Precision Oncology, Cancer Centre, Faculty of Health Sciences, University of Macau, Macau, Taipa, 999078, China; c Department of Chemistry, Korea University, Seoul 02841, South Korea; d Department of Cardiovascular Surgery, Zhongnan Hospital of Wuhan University, School of Pharmaceutical Sciences, Wuhan University, Wuhan 430071, China; e TCM Prevention and Treatment of Metabolic and Chronic Diseases Key Laboratory of Sichuan Province, Hospital of Chengdu University of Traditional Chinese Medicine, Chengdu 610075, China; f Innovative Institute of Chinese Medicine and Pharmacy/Academy for Interdiscipline, Chengdu University of Traditional Chinese Medicine, Chengdu 611137, China; g Department of Critical Care Medicine, Hospital of Chengdu University of Traditional Chinese Medicine, Chengdu 610075, China; h School of Pharmacy, Chengdu University of Traditional Chinese Medicine, Chengdu 611137, China

**Keywords:** NIR-II, Gas therapy, Phototherapy, Carbon monoxide, Nanoparticles

## Abstract

Endogenous gaseous molecules, especially carbon monoxide (CO), exhibit unique therapeutic potential in regulating cancer, infections, and inflammation owing to their dose-dependent bioactivity and multi-faceted mechanisms. However, challenges in spatiotemporal control and real-time monitoring of CO delivery hinder clinical translation. The integration of near-infrared II (NIR-II) imaging with CO nanodelivery systems provides a promising avenue for precise visualization and controlled gas therapy, offering deep tissue penetration, high resolution, and a favorable signal-to-noise ratio for guided therapy. This review systematically summarizes recent advances in NIR-II-guided CO nanoplatforms, focusing on design strategies, including polymers, core-shell structures, Metal-Organic Frameworks (MOFs), and peptide-modified systems, as well as their stimulus-responsive mechanisms across exogenous light, microenvironmental, and organelle-targeted delivery. Furthermore, we highlight synergistic therapeutic strategies combining CO gas therapy with photothermal, photodynamic, chemodynamic, and immunotherapy under NIR-II guidance, demonstrating enhanced therapeutic efficacy while minimizing systemic toxicity. Despite these advances, significant challenges remain, including the lack of subcellular-level CO monitoring, incomplete synergy between NIR-II imaging and CO bioactivity, and hurdles in clinical translation. This review aims to provide critical insights into the rational design of NIR-II-guided CO nanodelivery systems, offering a blueprint for their future development towards precise, safe, and effective disease treatment.

## Graphical abstract

This review summarizes recent advances in near-infrared II (NIR-II)-guided carbon monoxide (CO) nanomedicine, emphasizing design strategies, stimuli-responsive delivery, and synergistic therapies. It highlights how NIR-II imaging enables precise, controllable CO release with enhanced therapeutic efficacy and reduced toxicity, while outlining key challenges and future directions toward clinical translation.

**Figure:**
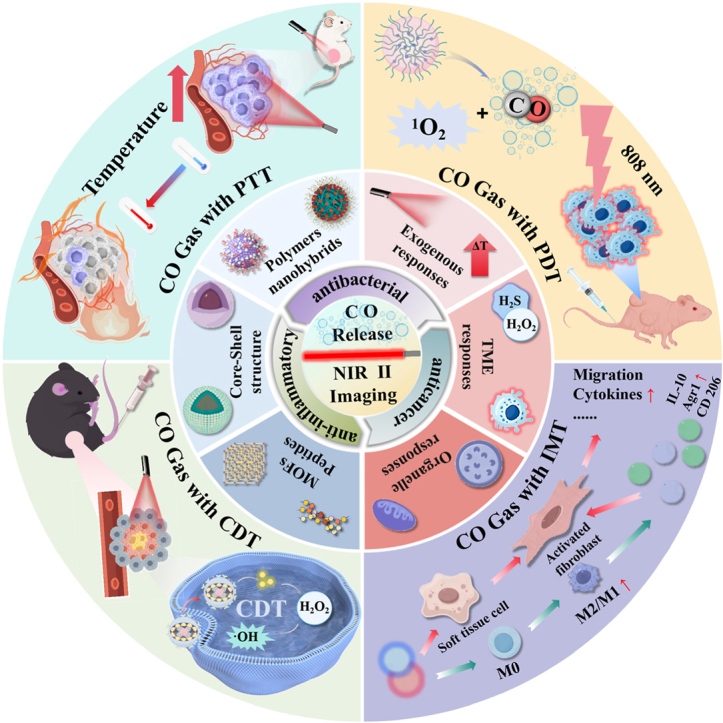

## 1 Introduction

In recent years, the role of endogenous gaseous signaling molecules in gas therapy for disease regulation has attracted increasing attention [[1], [2], [3]]. Among them, gaseous molecules such as CO, hydrogen sulfide (H_2_S), and nitric oxide (NO) play important roles in physiological processes, including cell signal transduction, inflammatory response, angiogenesis, and immune regulation [4,5] (see Table 1, Table 2).

**Table 1:** Summary of NIR-II guided CO nanodelivery system.

Name	Absorption (nm)	Emission (nm)	Applied	Diseases	Ref.
COT	500 nm	/	CO + PTT	Antibacterial	[65]
PBPTV@mPEG (CO)	810 nm	970 nm	CO + PTT	Cancer	[33]
CO-MON	782 nm	910 nm	CO + PTT + ICD	Cancer	[67]
TDF-CO	1000 nm	/	CO + PTT	Cancer	[57]
TTQ-MnCO NPs	1050 nm	1250 nm	CO + PTT	Cancer	[64]
AgPt@CaCO_3_-FA	557 nm	1101 nm	CO + PDT + ICD	Cancer	[51]
MMGD NPs	808 nm	1100 nm	CO + PTT	Cancer	[41]
Stealth NanoBomb	730 nm	960 nm	CO + PTT	Cancer	[40]
DSACGM NPs	1095 nm	/	CO + PTT + CDT	Cancer	[66]
PC/AC@GR@HA	730 nm	/	CO + PTT + TDT	Cancer	[34]
DCNPs	808 nm	1060 nm	CO + In situ bioimaging	Acute liver injury	[45]
PBF-g-CO/PEG	808 nm	1060 nm	CO + PDT	Cancer	[61]
Pd@PdCO-MOF	665 nm	/	CO + PTT	Cancer	[36]
TiN + Fe (CO)_5_+ALG	600 nm	/	CO + PTT	Cancer and Bacterial infections	[47]
TTCO-NPs	780 nm	1070 nm	CO + ICD	Rheumatoid arthritis	[52]

**Table 2:** CO-releasing prodrugs: Responsive mechanisms and therapeutic applications.

Name	Responsemode	Release rate	Loading material	Targeting mechanism	Disease	Ref.
PCOD585	ONOO^−^	24.05 ± 1.964 ppm	PLGA	Macrophage membrane-coated	MIRI	[70]
FL1/2/3	Light	80/314/250 ppm	OEP、PLGA	EPR	Cancer	[71]
CORMs	UV	260 ppm	LiLuF_4_:Ce^3+^ SCNPs、Tween-20	EPR	Cancer	[72]
OHF	US	15–29 ppm	OFlav OEGMA	Ultrasound-triggered	*S. aureus* infection	[73]
3-HF	Light	10–35 ppm	POEGMA、TPP	Local injection	SA	[74]
Fe_3_(CO)_12_	ROS	2.39 μM	DSPE-PEG_2000_	EPR	Cancer	[75]
Fe_3_(CO)_12_	Light、ROS	0.1–2.8 ppm	Croc-PEG5K	Microenvironment-targeted	OA	[76]
MnCO	ROS	6–40 ppm	HMPB、GS-01、PPG-13、RBC-4T1	Immune escape Homologous targeting	Cancer	[77]
MnCO	ROS	0.05–1.2 um	RM、HA	HA-CD44	Cancer	[78]
COD	Light	0.78–1.84 um	DSPE-mPEG_2000_	EPR	Glaucoma	[79]
3-HF	Light	1 μM	PEG_45_	Local injection	POCD	[80]
Fe_2_(CO)_9_	ROS	1.2 μM	PSP	Hydrogen bond targeting cfDNA	Sepsis	[81]
Mn_2_ [CO]_10_	ROS	1.2 μM	HA-SH	Microenvironment-targeted	Diabetic chronic wound	[82]
Na_3_Mo (CO)_3_(CNCH_2_CO_2_)_3_)	FeCl_3_	504.8 μL	3D Printing	Fe^3+^-responsive coordination targeting	Tendinopathy	[83]
Fe_3_(CO)_12_ FeCO-Si	X Ray、ROS	2.9 ± 0.1 Equivalent	SiO_2_	EPR Microenvironment-targeted	Cancer	[84]

Of particular note is CO, a dose-dependent endogenous gaseous signaling molecule. At low concentrations, CO exhibits low toxicity to normal cells while demonstrating multiple therapeutic potentials. As an endogenous key gaseous signaling molecule, CO exhibits significant dose-dependent bidirectional characteristics in its physiological and pathological functions: under physiological conditions, it can participate in regulating vascular smooth muscle relaxation, inhibiting abnormal platelet aggregation, and maintaining mitochondrial respiratory chain homeostasis, serving as an important mediator for maintaining normal bodily functions; whereas in pathological scenarios, low concentrations of CO can regulate the tumor microenvironment(TME), disrupt bacterial energy metabolism pathways, and downregulate the expression of pro-inflammatory factors such as tumor necrosis factor-α (TNF-α) and interleukin-6 (IL-6), showing unique therapeutic potential in cancer, antibiotic-resistant bacterial infections, and chronic inflammatory diseases. In cancer therapy, CO can enhance anti-tumor immune responses. Meanwhile, its complex mechanism of action in the TME and potential therapeutic value have attracted extensive attention in the academic community; in antibacterial therapy, CO is capable of inhibiting the activity of multiple pathogenic bacteria, including drug-resistant strains. Its inhibitory effect is achieved by disrupting bacterial energy metabolism and biofilm architectures; simultaneously, in inflammatory regulation, CO can downregulate the expression of pro-inflammatory cytokines, such as TNF-α and IL-6, thereby alleviating local inflammatory damage. This property provides a novel research direction for the coordinated resolution of cancer, infection, and inflammation-related problems [[6], [7], [8]]. While CO exhibits prominent therapeutic potential, other endogenous gaseous molecules like NO and H_2_S also play roles in disease regulation, with distinct mechanisms: NO mainly exerts vasodilatory and antiplatelet effects, but its short half-life (∼3–5 s) and high reactivity easily lead to off-target toxicity; H_2_S can modulate cellular redox balance, yet its strong reducing property may disrupt normal cell metabolism at high concentrations. By contrast, CO has a longer *in vivo* half-life (∼4–6 h) and dose-dependent bioactivity—low concentrations specifically target diseased cells (e.g., tumor mitochondria, bacterial biofilms) while sparing normal tissues, making it more suitable for long-term controlled delivery via nanosystems. Cancer, as a major disease posing a severe threat to human health, has always been a focal point in medical research [9]. Despite the fact that traditional treatment modalities, such as surgery, chemotherapy, and radiotherapy, can control tumor progression to a certain extent, issues, including tumor cell heterogeneity, drug resistance, and toxic side effects on normal tissues, still present significant challenges to cancer therapy. Seeking new methods that can overcome these limitations and achieve precise anti-tumor effects has become an important direction in current research — as an endogenous gaseous signaling molecule with dose-dependent properties, CO exhibits multidimensional regulatory effects in cancer treatment, precisely providing a potential solution to this need. In cancer therapy, CO exhibits multidimensional regulatory effects [10]. CO can inhibit tumor cell proliferation by regulating the cell cycle, inducing endoplasmic reticulum stress, and activating autophagy, thereby affecting the antitumor activity of T cells. In addition to inhibiting tumor development from the ‘source of proliferation’, CO can further block cancer cell survival by regulating apoptosis-related pathways — specifically, it can induce apoptosis through two key mechanisms: It can also promote cell apoptosis by activating the Mitogen-Activated Protein Kinase (MAPK)/Extracellular Signal-Regulated Kinase 1/2 (Erk1/2) pathway and regulating B-cell lymphoma 2 (Bcl-2) family proteins [11,12]. Furthermore, CO can regulate epithelial-mesenchymal transition to inhibit tumor metastasis, influence tumor angiogenesis, and interact with the TME to exert antitumor effects by regulating the maturation and polarization of immune cells and alleviating hypoxia. The above effects mostly focus on the overall cells or the macroscopic TME, while the antitumor advantages of CO also manifest in more precise subcellular interventions — specifically, the targeted action on the mitochondria of cancer cells. Simultaneously, CO can specifically target the mitochondria of cancer cells, affect mitochondrial activity, and achieve a targeted attack on cancer cells [13,14]. These unique roles of CO in cancer treatment not only distinguish it from traditional therapies, but also, compared to other endogenous gaseous signaling molecules like H_2_S and NO, demonstrate irreplaceable advantages — these advantages are mainly reflected in two core aspects: Compared with gaseous molecules such as H_2_S and NO, CO has unique advantages in cancer therapy. In terms of microenvironment regulation, its selectivity in regulating tumor angiogenesis, controllability in inducing tumor cell apoptosis, and potential in activating antitumor immunity are all superior to those of NO [[15], [16], [17], [18]]. At the metabolic regulation level, the clear targeting effect of CO on the mitochondrial respiratory chain and its improvement of the hypoxic microenvironment makes its mechanism of action clearer than that of H_2_S.

Meanwhile, bacterial infections (especially chronic infections caused by drug-resistant bacteria) and their accompanying inflammatory responses (e.g., periodontitis and chronic wound infections) have also become intractable clinical problems because of their tendency to cause prolonged illness and aggravated tissue damage [19,20]. These infections not only disrupt the local tissue microenvironment but may also affect systemic homeostasis through the continuous release of inflammatory factors, further complicating treatment. Beyond cancer therapy, CO also shows significant value in antibacterial and inflammatory regulation. In the field of antibacterial therapy, CO can exert inhibitory effects on a variety of pathogenic bacteria (including drug-resistant bacteria) by disrupting bacterial energy metabolism and biofilm structures, especially showing prominent effects on stubborn infections with biofilm formation. In inflammatory regulation [21,22], CO can downregulate the expression of pro-inflammatory factors such as TNF-α and IL-6, while upregulating the levels of anti-inflammatory factors, thereby reducing local inflammatory responses [[23], [24], [25]]. For example, in diseases such as periodontitis and chronic wound inflammation, CO alleviates tissue damage and promotes repair (Scheme 2).

In addition, the flexibility of CO in administration methods, including the targeted design of CO-releasing molecules, safety control, and potential for synergistic enhancement when combined with other therapeutic approaches, enables it to show broad prospects in the treatment of tumors, infections, and inflammation-related diseases [26,27]. The necessity of developing new CO nanodelivery materials can be clarified by the shortcomings of existing clinical materials: Titanium alloys, due to their elastic modulus being very different from that of human bone, can easily cause ‘stress shielding’, and postoperative bone loss and infection risks are relatively high; Poly(methyl methacrylate) bone cement is non-degradable, prone to loosening over time, and the heat generated during polymerization can damage tissues, while postoperative infections are prone to recur. These traditional materials lack an integrated ‘treatment-support-safety’ function. In contrast, CO nanocarriers such as MOFs and polymers can precisely release CO to fight infection and regulate inflammation, while also optimizing biocompatibility and degradability, perfectly addressing the above disadvantages. This is exactly the core significance of developing such new materials [[28], [29], [30]].

Nevertheless, the accurate administration of CO gas, visual surveillance, and effectiveness assessment remain unresolved issues [31]. To address these challenges, nanodelivery systems and imaging technologies have been explored. NIR-II imaging technology offers a novel approach owing to its exceptional tissue penetration depth, minimal scattering impact, and superior signal-to-noise ratio [32]. Notably, NIR-II-guided CO nanomedicine differs fundamentally from traditional gas therapy and other light-controlled systems: compared with traditional CO gas therapy (e.g., inhaled CO or injectable small-molecule CO donors), it avoids the uncontrollable CO release (which causes systemic toxicity) and lack of real-time monitoring—relying on NIR-II's deep tissue imaging, it realizes “visualized and on-demand release” of CO at the lesion site; compared with other light-controlled systems (e.g., NIR-I or UV-triggered ones), NIR-II's longer wavelength (1000–1700 nm) enables ∼ 5-10 x deeper tissue penetration and lower photon scattering, solving the problem that shallow light cannot reach deep lesions (e.g., pancreatic tumors or deep-seated infections), while reducing photodamage to normal tissues. In recent years, CO-based nano-delivery systems have made significant progress in tumor therapy, particularly in the structural design of their integrated platforms. Advances in nanoengineering technology have facilitated the fabrication of CO-based nanoplatforms with excellent biocompatibility, controlled CO release, and high targeting efficiency, paving the way for their potential clinical translation.

Compared with previously reported reviews on CO gas therapy, this review focuses on the deep integration of NIR-II imaging and CO nanodelivery systems. The core differences and features are reflected in three aspects: First, it systematically summarizes the technical advantages specifically related to the NIR-II window (deep tissue penetration, high signal-to-noise ratio), rather than broadly discussing gas therapy or light-controlled delivery. Second, it emphasizes the association between fundamental material structure design and functional optimization along with the synergistic adaptability of the NIR-II response mechanism, filling the gap in existing reviews regarding the detailed coverage of integrated “imaging–delivery–release” design. Third, it strengthens the analysis of subcellular targeting (mitochondria, lysosomes) and multimodal synergistic therapy (PTT/PDT/CDT), providing more specific technical references for precision therapy.

**Scheme 1:**
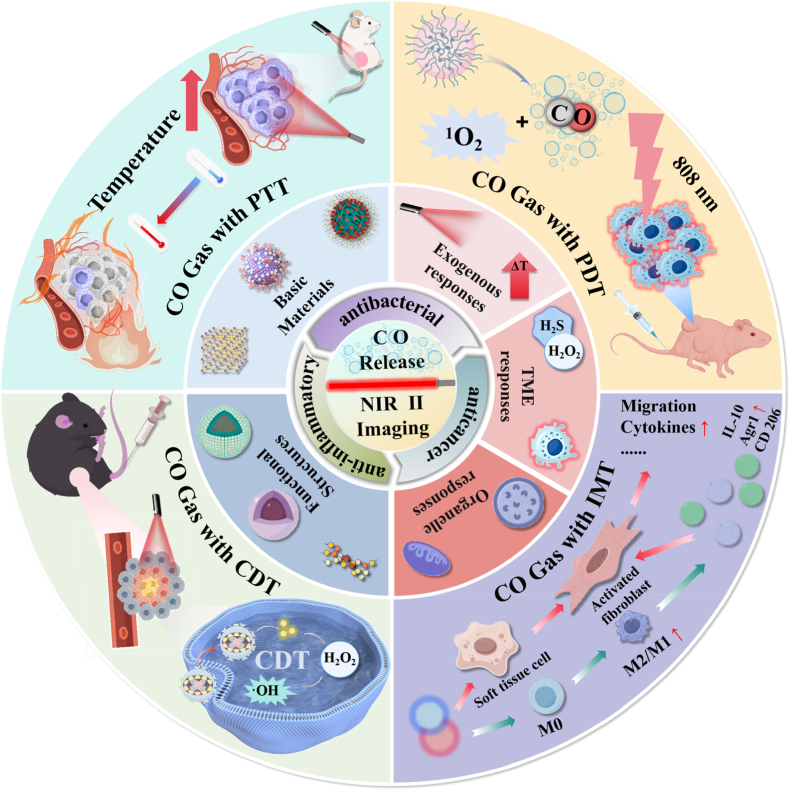
NIR-II for biomedical applications: Structural design of CO nanosystems, response patterns, and combination therapy with photothermal therapy (PTT), photodynamic therapy (PDT), chemodynamic therapy (CDT), and immunotherapy (IMT).

This mini-review provides an in-depth analysis of NIR-II-guided CO nanoplatform delivery systems. Next, we explore the structural design of their loaded platforms, various stimulus-response modes, and the mechanisms and combination therapeutic applications of CO nanoplatform delivery systems in photothermal therapy (PTT), photodynamic therapy (PDT), chemodynamic therapy (CDT), and immunotherapy (IMT) (Scheme 1).

**Scheme 2:**
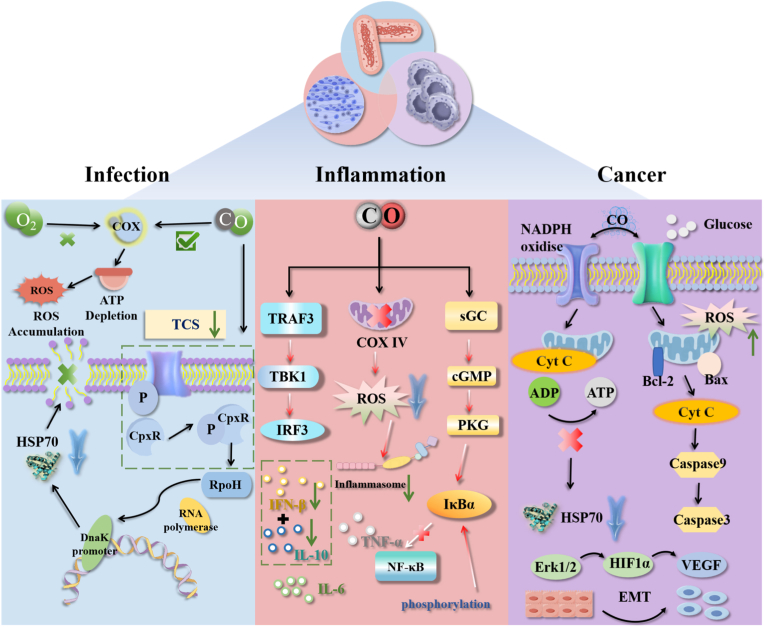
Schematic Diagram of Carbon Monoxide's Mechanisms in Antibacterial, Anti-Infective, and Anticancer Effects.

## 2 CO delivery carriers: basic materials & functional structures

Precise delivery and controlled release of CO are at the core of NIR-II guided gas therapy, and the rational design of nanocarriers is the key prerequisite for achieving this goal — these carriers not only need to efficiently load CO donors and integrate NIR-II imaging/photothermal functions, but also require good biocompatibility and targeting capabilities to overcome biological barriers such as clearance during blood circulation and tumor matrix penetration. This section focuses on the selection of basic materials and functional structure optimization of CO nanocarriers. It first introduces the characteristics and applications of basic carriers such as polymers, nanohybrids, and MOFs, and then elaborates on how enhancement systems such as peptide modification and core-shell structures further address the limitations of basic carriers, laying a structural foundation for the subsequent discussion on responsive modes and combination therapies.

### 2.1 Polymers、Nanohybrids and MOFs: Basic carriers for CO delivery

As the basic carriers of CO nanodelivery systems, polymers, nanohybrids, and MOFs have become key choices for achieving efficient CO loading and controlled release due to their respective unique structural advantages. Polymers and nanohybrids can form stable structures through molecular self-assembly and easily integrate NIR-II imaging and photothermal functions; MOFs, on the other hand, rely on their porous characteristics and tunable ligand structures to accommodate CO donors of different sizes, providing foundational support for subsequent responsive modes and synergistic therapies. This section will elaborate on the design principles, performance features, and specific applications of nanodelivery systems based on these three types of basic carriers in CO delivery.

**Fig. 1:**
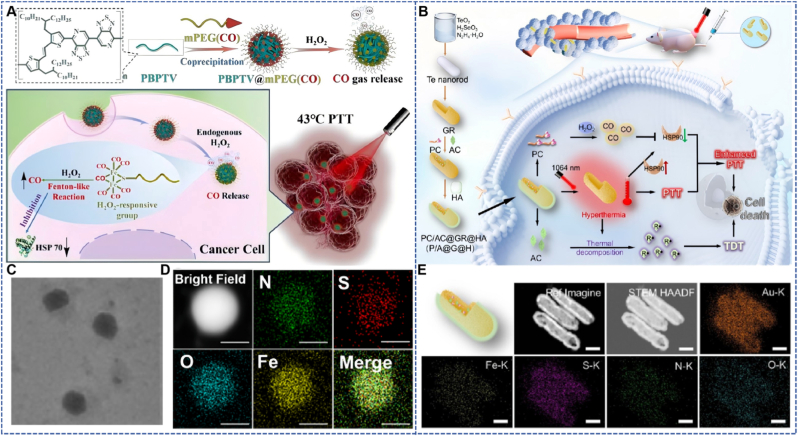
(A) Nano-hybrid particles for CO delivery and synergistic enhancement of low-temperature photothermal therapy in breast cancer [33]. Reprinted from with permission from Wiley. (B) Gold nanorods for CO delivery-low-temperature photothermal-pyrodynamic synergistic therapy of colon cancer [34]. Reprinted from with permission from ACS Publications. (C) The particle size measured by TEM. Scale bar 100 nm. (D) The EDS elemental mapping of nanobomb. Scale bar 50 nm. (E) Model image, ref image, STEM image, and the corresponding element mapping images of P/A@G@H (scale bar 50 μm). (For interpretation of the references to color in this figure legend, the reader is referred to the Web version of this article.)

The nanobomb developed by Ma et al. is a nanohybrid formed *via* the self-assembly of the linear NIR-II Aggregation-Induced Emission (AIE) polymer PBPTV and the CO carrier polymer mPEG (CO) (Fig. 1A) [33], with a size of approximately 71 nm. This nanobomb can release CO gas triggered by overexpressed H_2_O_2_ in the TME. Traditional PTT has limitations, including thermal tolerance and adverse reactions. Low-temperature PTT is an emerging therapeutic strategy; however, its efficacy is often significantly compromised by the upregulation of heat shock proteins (HSPs). CO gas can significantly inhibit the expression of HSPs, thereby enhancing the antitumor efficiency of low-temperature PTT. Experimental results show that the nanohybrid possesses excellent photothermal conversion efficiency: under 808 nm laser irradiation, the temperature can rise to 76.5 °C within 3 min, with a photothermal conversion efficiency as high as 38.1 %. In the 4T1 tumor-bearing mouse model, the nanohybrid combined with CO therapy and low-temperature PTT exhibited efficient tumor suppression, with continuous reduction in tumor volume. During treatment, there was no significant change in the body weight of the experimental mice, indicating biosafety.

In the PBPTV@mPEG (CO) nanohybrids designed by Ma et al., the molecular structure of the linear NIR-II AIE polymer PBPTV is key to its high photothermal conversion efficiency of 38.1 %: PBPTV uses benzodithiophene (BDT) as the electron donor and thienothiophene (TT) as the conjugated bridging unit, forming a large π-conjugated system that broadens the absorption spectrum into the NIR-II region (810 nm absorption peak) to match the 808 nm laser; its strong electron-withdrawing groups on the molecular chain (such as cyano groups) can regulate intramolecular charge transfer (ICT), reduce fluorescence radiative dissipation, and facilitate non-radiative transitions, achieving efficient conversion of laser energy into heat. Furthermore, the AIE property of PBPTV prevents aggregation-caused quenching during self-assembly, further maintaining high photothermal efficiency and providing energy support for CO-assisted low-temperature photothermal therapy(LPTT).

The P/A@G@H nanocapsule designed by Tang et al. is composed of gold hollow nanorods (GR), CO storage polymer mPEG (CO), and free radical initiator 4,4′-azobis (4-cyanovaleric acid), with hyaluronic acid (HA) modified on the surface to achieve active targeting. This nanocapsule can generate heat under the excitation of the NIR-II laser, inhibit the expression of HSPs *via* CO, and realize thermodynamic therapy (TDT) through thermal decomposition of free radical initiators (Fig. 1B) [34]. Although PTT has potential in tumor treatment, the application of single PTT has limitations, such as upregulation of HSPs leading to thermal tolerance of tumor cells, and insufficient heat diffusion resulting in the survival of tumor cells. In addition, traditional PTT is difficult to achieve long-term cytotoxic effects. Combining with TDT can make up for these deficiencies and achieve a synergistic therapeutic effect. Experiments demonstrate that P/A@G@H nanocapsules exhibit good biocompatibility and multimodal synergistic therapeutic effects both *in vitro* and in vivo. In the CT26 tumor-bearing mouse model, compared with the control group, the tumor volume in the P/A@G@H combined with laser irradiation group was significantly reduced, indicating its excellent tumor-killing effect. During the treatment, there was no significant change in the body weight of the mice, suggesting good biosafety of the material. Observations *via* H&E staining and TUNEL staining showed that the tumor tissue damage in the combined treatment group was the most severe, while normal tissues were not significantly affected.

However, although polymer and nanohybrid systems show good versatility in CO delivery, their structural uniformity and limited stimulus response accuracy directly restrict the precise spatiotemporal regulation of CO release. In order to break through this limitation, the core-shell structure nanocarrier realizes precise functional partitioning through hierarchical design—the core layer can efficiently load therapeutic components such as CO donors, and the shell layer can integrate surface functions such as targeting molecules and stabilizing groups, which not only significantly optimizes the release kinetics of CO, but also improves the stability of the system *in vivo* circulation, becoming an important technological breakthrough to solve the above problems.

MOFs possess unique two-dimensional porous structures. CO nanodelivery platforms designed based on this structure can provide favorable conditions for the controlled release of CO gas and significantly optimize its release kinetic properties [35]. Although the high loading capacity of MOFs is directly related to their relatively large specific surface area, when it comes to different biomacromolecules such as proteins and siRNA, the regulation of pore size, surface functional groups, and metal coordination points plays a more critical and differential role in loading efficiency and stability: For large biomacromolecules like proteins (usually several to tens of nanometers in size), the carrier pore size needs to precisely match the molecular size — Qian Qianjun's team significantly increased the loading of certain proteins by adjusting the pore size of mesoporous silica, while also reducing stability loss caused by macromolecular aggregation. This approach equally applies to MOF carriers, meaning the pore size needs to be designed by adjusting the ligand length or metal node spacing. For small nucleic acids such as siRNA, the role of surface functional groups is more prominent. For example, natural algal carriers, with abundant surface hydroxyl and carboxyl groups, can form stable complexes with siRNA via electrostatic interactions, preventing its degradation by nucleases. Similarly, MOF surfaces can be modified with amino or phosphate groups to enhance interactions with nucleic acid molecules. Regarding metal coordination points, some previous studies provide useful insights. Traditional MOF metal nodes may nonspecifically bind biomacromolecules, affecting their activity. By using thiol coordination to replace metal nodes, the porous structure of the MOF is retained, while specific coordination reduces interference with protein and siRNA activity and improves the stability of the complex in physiological environments after loading. Based on this, the design principles of MOF carriers for different biomacromolecules can be summarized as follows: match pore size to molecular dimensions, modify specific functional groups to enhance interactions, and optimize coordination methods to minimize interference with biological activity, thus achieving efficient and stable loading.

**Fig. 2:**
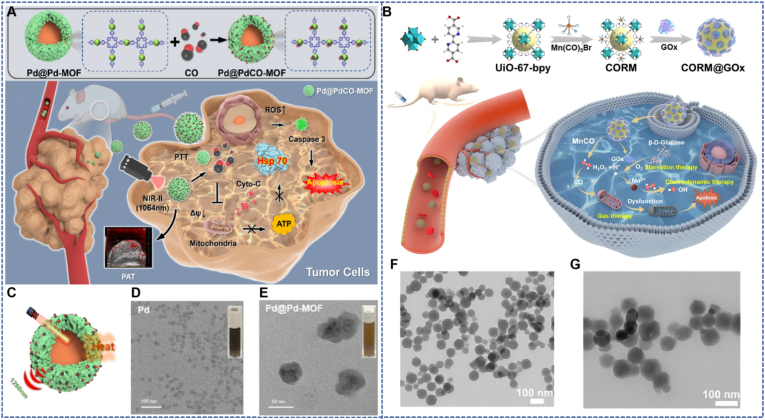
(A) Two-dimensional planar MOFs nanosheets for the synergistic enhancement of CO delivery and PTT [36]. Reprinted from with permission from Elsevier. (B) Schematic illustration of the synthesis of a MOF-based H2O2-responsive CO-releasing nanoplatform and its application in synergistic starvation/chemodynamic/gas combination therapy for cancer [37]. Reprinted from with permission from Elsevier. (C) Schematic illustration of the photothermal conversion of Pd@Pd-MOF NPs. (D–E) Transmission electron microscope image of Pd and Pd@Pd-MOF dispersions. (F–G) TEM images of UiO-67-bpy and CORM@GOx.

Yao et al. designed HA-modified Pd@PdCO-MOF nanoparticles, with a core consisting of 20–30 nm Pd nanosheets and a surface coated with a porphyrin-palladium MOFs (Pd-MOF) shell layer. The two-dimensional layered MOF is formed by the coordination of sodium tetrachloropalladate and tetrapyridylporphyrin (TPyP) at a molar ratio of 2:1. After reduction to zero-valent Pd with hydrazine, CO is loaded. The outer layer forms a hydrophilic layer with a thickness of approximately 10 nm through covalent bonding between the carboxyl groups in HA molecules and the hydroxyl groups on the surface of Pd-MOF. HA modification reduces the zeta potential of the nanoparticles from +25 mV to −15 mV and achieves active targeting through specific binding to the CD44 receptor on the surface of tumor cells (dissociation constant Kd = 1.2 μM). The hydrodynamic diameter measured by dynamic light scattering is approximately 157 ± 10 nm. To address the issue of thermal tolerance in PTT for deep tumors, this design utilizes the advantage of the NIR-II optical window (1000–1700 nm) with a penetration depth of up to 10 mm. The photothermal effect of Pd nanosheets under 1064 nm laser (with a conversion efficiency of 44.6 %) triggers the rapid release of CO from Pd-MOF (14 μM/60 min). CO enhances the sensitivity of tumor cells to photothermal effects by inhibiting the heat shock protein HSP90; the active targeting property of HA modification can reduce the distribution of nanoparticles in normal tissues, with the accumulation in organs such as the liver and spleen being 40 % lower than that of the control group. Meanwhile, the photoacoustic signal of the porphyrin ligand in Pd-MOF at 1260 nm enables imaging guidance, forming a synergistic system of “photoacoustic imaging-photothermal-CO therapy” (Fig. 2A) [36]. *In vitro* experiments confirmed that under 1064 nm laser irradiation (1 W/cm^2^), Pd@PdCO-MOF releases 3.9 μM CO, the Reactive Oxygen Species (ROS) production reaches 1.8 μM, and the cell death rate reaches 92 %; in the 4T1 tumor-bearing mouse model, after intravenous injection of Pd@PdCO-MOF, at the optimal enrichment time point of 8 h, a significant temperature increase of 12.9 °C in the tumor area was observed under photoacoustic imaging guidance; continuous observation until day 12 showed that the tumor volume in this group only increased by 1.7 times, while that in the control group increased by 12.1 times, and tumors in two-thirds of the mice completely regressed. The hemolysis rate was <1 %, and H&E staining of major organs and blood biochemical indicators were normal, confirming the high efficiency and low toxicity of this nano-platform.

Yang et al. used a Zr(IV)-based metal-organic framework (UiO-67-bpy) as a carrier to design a CORM@GOx nanoplatform capable of cascade reactions, for H_2_O_2_-responsive controllable CO release and synergistic anticancer therapy. This MOF was synthesized via a solvothermal method. Its porous structure not only efficiently encapsulates the CO donor manganese carbonyl (MnCO) and glucose oxidase (GOx), but also, through the bipyridine groups in the ligand, forms coordination with MnCO, enhancing the stability of CO donor loading. The GOx loading reaches 18 wt%, and the MOF's crystal structure remains intact after loading (Fig. 2B) [37].

Functionally, once the nanoplatform enters tumor cells, GOx first catalyzes endogenous glucose to produce gluconic acid and H_2_O_2_: the former lowers the TME pH and cuts off cellular energy supply to achieve starvation therapy, while the latter acts as a trigger signal to induce MnCO to release CO via a Fenton-like reaction and generate Mn^2+^; Mn^2+^ further reacts with H_2_O_2_ to produce highly toxic •OH, enhancing the effect of CDT. In vitro experiments showed that in the presence of glucose, CORM@GOx significantly increases ROS levels in HeLa cells, with a cell death rate up to 93 %. Hemoglobin assay results demonstrated that under conditions of 40 μM H_2_O_2_, the CO release concentration reached 3.4 μM within 40 min, and the platform remained stably dispersed in PBS and 10 % FBS after 4 days of storage.

In animal experiments, HeLa tumor-bearing mice treated with CORM@GOx showed efficient tumor accumulation via the enhanced permeability and retention (EPR) effect, peaking at 12 h. The tumor inhibition rate after treatment reached 94.3 %, far superior to the individual UiO-67-bpy group (no significant inhibition) and CORM group (60.3 % inhibition). H&E staining and TUNEL assays revealed obvious apoptosis features in tumor tissues, while the mice's body weight remained unchanged and major organs showed no pathological damage, confirming that this MOF-based nanoplatform achieves CO gas-starvation-CDT synergistic therapy while exhibiting good biocompatibility.

The performance differences of various base carriers stem from structural adaptability: polymer carriers rely on FDA-approved materials (such as PLGA), providing advantages for clinical translation, but their poor structural uniformity results in fluctuating release; MOFs, with their porous structure, can accommodate diverse CO donors, yet the *in vivo* accumulation risk of some metal nodes lacks sufficient verification, making them clinically controversial; nano-hybrids achieve high efficiency through photothermal-CO synergy, but their *in vivo* stability is greatly affected by the physiological environment. In terms of translational potential, polymer carriers have an advantage due to abundant toxicological data, while MOFs need to prioritize addressing the controllable release of metal ions.

In summary, polymers and nanohybrids achieve efficient integration of CO donors with NIR-II imaging, photothermal, and other functional modules through molecular self-assembly, while MOFs, relying on their unique porous structures and flexible coordination control, effectively address key issues in CO loading stability and release kinetics optimization. These three types of fundamental carriers each play to their strengths, collectively providing a solid functional foundation for precise CO delivery. However, in practical applications, these basic carriers still face real challenges such as insufficient targeting accuracy and limited stimulus response specificity. Enhanced systems composed of peptide modifications and core-shell structures are precisely capable of addressing these shortcomings. Based on this, the next section will focus on the design logic, performance advantages, and specific applications of such enhanced systems in CO delivery.

### 2.2 Peptide modifications、Core-Shell structures: CO delivery enhancement systems

In response to issues such as insufficient targeting of basic carriers and suboptimal stability *in vivo*, peptide modification and core-shell structures have emerged as two key enhancement systems. Peptide modification achieves active targeting through the high affinity of polypeptides for specific receptors, while core-shell structures optimize carrier stability and stimulus response efficiency through functional compartmentalization. Both approaches can significantly improve the precision and reliability of CO delivery. This section will explore the structural design logic, performance advantages, and practical effects of these two enhancement systems in CO delivery.

**Fig. 3:**
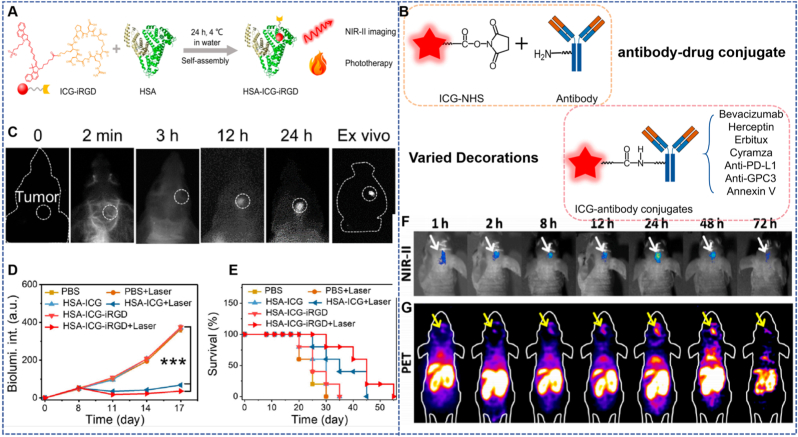
(A) Subsequent conjugation of the HSA-ICG intermediate with the iRGD peptide, enabling application in glioma [38]. (B) Construction of the HSA-ICG-iRGD probe through encapsulation of ICG into the cavity of HSA [39]. Reprinted from with permission from Science and Technology Review Publishing House. (C) *In vivo* and *ex vivo* NIR-II imaging of HSA-ICG-iRGD in an orthotropic U251 glioma tumor model. (D–E) Bioluminescence intensity and survival curves of U251 glioma-bearing mice with different treatments. (F–G) *In vivo* NIR-II and PET imaging at different time points after ^64^Cu-DOTA-FA-ICG injection.

The excellent targeting property of peptide-based drugs forms a highly efficient synergistic effect with CO gas therapy. This combination not only achieves complementary advantages in functions but also promotes the structural design of CO nanodelivery platforms to evolve towards a more perfect direction. After discovering the tail emission property of ICG in the NIR-II window, Carr et al. successfully constructed Bev-ICG conjugates through amine-ester condensation reaction by covalently coupling N-hydroxysuccinimide ester of ICG (ICG-NHS) with lysine residues of antibodies such as bevacizumab (Bev). In this conjugate, each antibody molecule is coupled with 2–3 ICG molecules, with a particle size of approximately 150–200 nm. Considering that ICG itself lacks targeting property and has poor stability, while Bev has a high specific binding ability to VEGF, the design of Bev-ICG is based on this property (Fig. 3B). Experimental results showed that the binding efficiency of Bev-ICG to HCT 116 colorectal cancer cells was as high as 85 %, significantly higher than that of the IgG-ICG control group (only 12 %); in NIR-II endoscopic imaging of tumor-bearing rats, Bev-ICG made the tumor Signal-to-Background Ratio (SBR) reach 15, which not only clearly displayed blood vessels with a diameter >20 μm but also improved the NIR-II imaging signal-to-noise ratio to 15:1, achieving accurate delineation of tumor boundaries [38].

Cheng et al. embedded ICG in the hydrophobic cavity of human serum albumin (HSA) to make it self-assemble into 50–80 nm nanoparticles, and then constructed HSA-ICG-iRGD probes by coupling iRGD peptides. Among them, iRGD can enhance targeting by recognizing αvβ3 integrin, and HSA embedding can extend the half-life of ICG from 3 min to 2.5 h. With the endogenous biocompatibility of HSA and the active targeting property of iRGD, this probe can effectively break through the tumor stromal barrier, especially suitable for deep lesions such as brain tumors; meanwhile, HSA embedding can avoid the aggregation-caused quenching of ICG, improve the photothermal conversion efficiency to 38 %, and thus enhance the effect of low-temperature PTT (Fig. 3A). Experimental results showed that the uptake of HSA-ICG-iRGD by U251 glioma cells was 2.3 times that of HSA-ICG; in the glioma model, it achieved a tumor inhibition rate of 91.1 %, and H&E staining results of major organs of mice were normal, fully confirming the safety and effectiveness of this probe [39].

**Fig. 4:**
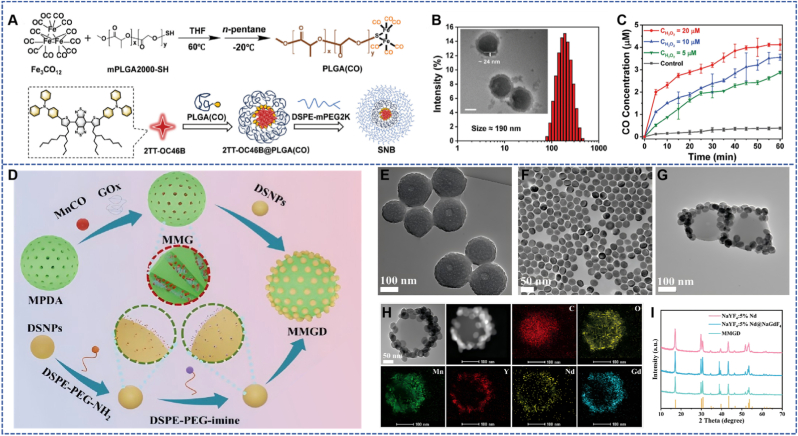
(A) Core-shell structured particles for CO delivery and synergistic enhancement of LPTT in pancreatic cancer [40]. Reprinted from with permission from Wiley. (B) Size distribution and TEM of SNB. (C) The release rate of CO with the concentration changes of H_2_O_2_. (D) Hierarchically coated MMGD nanoparticles for acid-responsive switch-mediated PTT and NIR-II imaging of lung cancer microenvironment [41]. Reprinted from with permission from Wiley. (E) TEM images of MPDA nanoparticles. (F) TEM images of NaYF_4_:5 %Nd@NaGdF_4_ DSNPs. (G) TEM images of MMGD nanoparticles. (H) TEM image and corresponding elemental mapping of MMGD, showing the spatial distribution of C, O, Mn, Y, Nd, and Gd elements. (I) Powder X-ray diffraction (XRD) patterns of NaYF_4_:5 %Nd, NaYF_4_:5 %Nd@NaGdF_4_, and MMGD.

Core-shell structured nanocarriers are a class of nanosystems that achieve functional partitioning and synergistic regulation through hierarchical design. Their core layer can load therapeutic molecules (such as CO donors), while the outer shell layer endows them with targeting ability, stability, and stimulus responsiveness, thus exhibiting unique advantages in the field of CO delivery. The “Stealth NanoBomb (SNB)” developed by Ma et al. forms a core layer *via* the self-assembly of small-molecule NIR-II AIEgens (2 TT-OC46B) and CO carrier polymer PLGA (CO), with the outer layer coated with PEG-lipids to form a shell structure [40]. The overall morphology is spherical (approximately 151.5 nm), with a total core-shell size of about 175.5 nm and an average shell thickness of 24 ± 0.6 nm. In this design, the core layer loads the CO donor PLGA (CO) and provides NIR-II imaging signals, while the PEG-lipid shell endows it with “stealth” properties to reduce clearance during blood circulation. Overexpressed H_2_O_2_ in the tumor microenvironment can trigger CO release from the core layer, inhibit the expression of HSPs, and enhance the efficacy of moderate-low temperature PTT. Under 808 nm laser irradiation, the photothermal conversion efficiency reaches 33.1 %, and the performance remains stable after 6 heating-cooling cycles. It achieves efficient tumor suppression in pancreatic cancer mouse models with no significant toxicity (Fig. 4A).

The MMGD nanoplatform constructed by Zhou et al. adopts a core-shell structure, using mesoporous polydopamine (MPDA) as the core to load CO prodrug MnCO and GOx [41]. The surface is coated with DSPE-PEG-imine-modified lanthanide-doped down-shifting luminescent nanoparticles (DSNPs) as the shell layer. In this design, DSNPs not only serve as a NIR-II fluorescence imaging probe but also act as an acid-unlocked gating switch. The slightly acidic TME can cause DSNPs to dissociate, thereby exposing GOx and releasing MnCO. The catalytic reaction of GOx produces H_2_O_2_ and creates a more acidic environment, promoting in situ CO generation, which reduces cytochrome *c* oxidase activity and adenosine triphosphate (ATP) levels to induce mitochondrial damage. Meanwhile, MPDA has NIR light absorption capability for PTT. The photothermal conversion efficiency of MMGD is 41.7 %, and under 808 nm laser irradiation (1 W cm^−2^), the temperature of an 800 μg mL^−1^ MMGD solution can rise to 46.2 °C within 10 min. *In vitro* experiments show that the late apoptosis rate of A549 cells in the MMGD (pH 6.5) + Laser group reaches 54.5 %. In A549 tumor-bearing mice, the MMGD + Laser group exhibits the best tumor inhibition effect, with the average tumor volume decreasing to less than 40 % during treatment, and no significant damage to major organs is observed, confirming its safety (Fig. 4D).

The core carriers of current CO nanodelivery systems each have their own characteristics: polymer carriers have good biocompatibility and can achieve passive targeting easily, but they have poor structural uniformity and insufficient precision in stimulus response; MOF carriers have high specific surface area and adjustable pore size, making them adaptable to CO donors of different sizes, but some metal nodes are relatively toxic and their controllable degradation *in vivo* needs improvement; core–shell structures have clear functional partitioning and can integrate multimodal therapeutic capabilities, but their preparation process is complex and yield is low; peptide-modified systems have outstanding targeting accuracy (such as iRGD and HA modifications enabling active targeting), but peptide chains are easily degraded by proteases, resulting in weak *in vivo* circulation stability. From the perspective of clinical translation potential, polymers have a stronger foundation because some materials (like PLGA) have already been approved by the FDA. MOFs and core–shell structures need to prioritize solving safety and scalable preparation issues, while peptide-modified systems need to enhance their anti-degradation capability.

The core difference between peptide modification and core-shell structures lies in the trade-off between ‘targeting precision and preparation complexity’: Peptide modification (such as iRGD) enhances targeting efficiency through receptor-specific binding, but the peptide chains are easily degraded by proteases, and their actual effectiveness in complex TME remains controversial; core-shell structures achieve functional compartmentalization and integration, but face issues of low production yield and significant batch-to-batch variability, and the long-term immunogenicity of PEG-lipid shells needs further evaluation.

This chapter focuses on the core carrier design of CO nanodelivery systems, systematically summarizing the characteristics and applications of two key types of carriers: on one hand, polymers, nanohybrids, and MOFs serve as basic carriers, achieving efficient CO loading and controlled release through molecular assembly and porous structures. Some carriers integrate materials such as gold nanorods to impart photothermal conversion capabilities, laying the functional foundation for subsequent combination therapies; on the other hand, peptide modifications and core-shell structures form enhanced systems. Leveraging the targeting specificity of peptides and the functional compartmentalization of core-shell structures, these systems further improve carrier targeting accuracy, *in vivo* circulation stability, and stimulus-responsive efficiency. The design of these two types of carriers collectively addresses the core challenges of traditional CO delivery–insufficient loading, weak targeting, and poor release controllability–providing solid structural support for designing responsive mechanisms in delivery systems. The next section will focus on how to achieve precise, on-demand CO release based on these carrier characteristics through modes such as exogenous stimulation, endogenous microenvironment response, or organelle targeting.

## 3 Response modes of existing CO nanodelivery system

The design of response modes in existing CO nanodelivery systems primarily aims to achieve “on-demand and precise” release of CO at disease sites. Based on the source of triggering signals, these modes can be divided into three major categories, each complementing the others in terms of precision and applicability. The first category is exogenous light-stimulated responses, where NIR-II laser serves as the core trigger. Leveraging its non-invasive nature and spatiotemporal controllability, it can simultaneously facilitate CO release and imaging guidance, making it particularly suitable for superficial lesions or deep-seated lesions that can be targeted with a light source (e.g., gastrointestinal tumors assisted by endoscopy). The second category is endogenous microenvironment-responsive, triggered by unique physiological characteristics at disease sites (such as high H_2_O_2_ and acidic pH in TME, or high H_2_S concentrations at infection sites) to autonomously release CO without external intervention. This avoids the limitations of insufficient light penetration and is suitable for complex *in vivo* disease scenarios. The third category involves organelle-targeted precise responses, delivering CO to critical subcellular structures like mitochondria and lysosomes, thereby enhancing delivery precision from the tissue/cell level to the organelle level. This not only strengthens CO's specific cytotoxic effects on diseased cells (e.g., disrupting energy metabolism via mitochondrial targeting) but also reduces damage to normal organelles. This section will sequentially explain the design principles of these three response modes, the construction of representative nano-platforms, and their practical applications, analyzing the advantages and limitations of each to provide guidance for optimizing CO nanodelivery system response mechanisms and improving their clinical adaptability.

### 3.1 Exogenous light stimulus responses

Exogenous light-stimulated responses are one of the core modes of NIR-II guided CO delivery. Their main advantage lies in utilizing the spatiotemporal controllability of NIR-II lasers to simultaneously achieve CO release triggering and imaging guidance, making them particularly suitable for superficial lesions or deep lesions that can be targeted with endoscopic assistance. This section will focus on the design principles activated by NIR-II lasers, introduce the construction and applications of representative nanoplatforms, and analyze their advantages as well as the limitations posed by restricted tissue penetration depth.

**Fig. 5:**
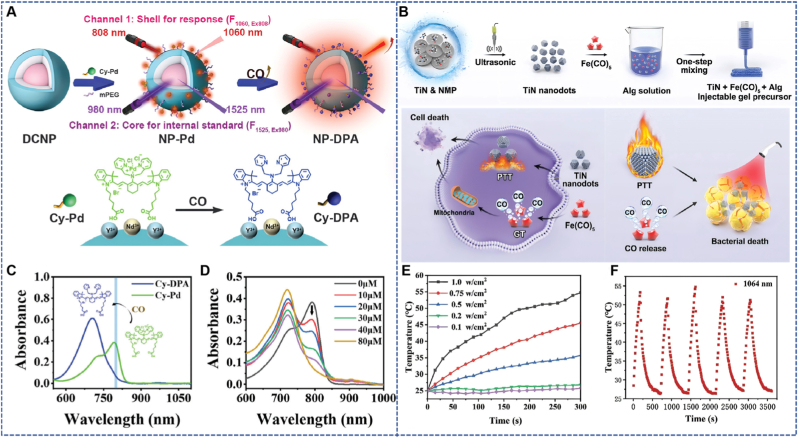
(A) Photoactivatable NIR-II ratiometric fluorescent NP-Pd for in situ bioimaging of CO [45]. Reprinted from with permission from Wiley. (B) Photoresponsive nanodots (NDs)-based multifunctional injectable hydrogel for simultaneous tumor therapy and antibacterial therapy [47]. Reprinted from with permission from Wiley. (C) Structural and absorption spectrum changes of Cy-Pd and Cy-DPA before and after CO release. (D) Changes in absorption spectra tested upon addition of CORM-2 (0–80 μM, in 30 s). (E) Photothermal heating profiles of TiN nanoparticles during exposure to a 1064-nm laser (0.75 W cm^−2^, 5 min) as a function of concentration. (F) Photothermal stability (5 laser on/off cycles) of TiNnanodots (Ti-NDs).

Phototherapy, with its significant advantages such as non-invasiveness and precision in the treatment area, has been successfully applied in CO nanodelivery systems based on exogenous light-dependent activation mechanisms [[42], [43], [44]]. Guo et al. designed a NIR-II fluorescent ratiometric nanoprobe (NP-Pd) that enables in situ bioimaging of CO relying on a light-activation mechanism. It uses downconversion nanoparticles (DCNPs) with a core-shell-shell (CSS) structure as the luminescent substrate, whose surface is modified with heptamethylcyanine-palladium complex (Cy-Pd) and methoxy polyethylene glycol (mPEG). The luminescence of DCNPs strictly depends on the activation by light of specific wavelengths, emitting NIR-II light at 1060 nm and 1525 nm under excitation by 808 nm and 980 nm lasers respectively. The core principle of this probe is based on the functional differentiation and anti-interference advantages of the dual channels: In the CSS-structured DCNPs used by the probe, the core emits 1060 nm fluorescence when excited by an 808 nm laser. Its surface-modified Cy-Pd complex can specifically bind with CO (CO causes the Pd^2+^ to dissociate via coordination, restoring the fluorescence of the Cy group, so the 1060 nm signal increases with the CO concentration). Meanwhile, the shell emits 1525 nm fluorescence when excited by a 980 nm laser. This channel is not modified with any CO-responsive groups, making its signal stable and useable as an internal reference to correct interference. Both channels operate within the NIR-II window (1000–1700 nm), with a low photon scattering coefficient (about 1/10 of NIR-I) and minimal tissue absorption, which reduces signal attenuation in deep tissue imaging. Furthermore, the significant wavelength difference (>400 nm) allows efficient signal separation to avoid crosstalk, ultimately ensuring high specificity and sensitivity for CO detection. This probe combines the light-activation advantages of NIR-II fluorescence imaging with the ratiometric fluorescence measurement method, improving detection accuracy by comparing signals from two independent fluorescence channels activated by different light sources. *In vitro* experiments showed that after activation by 808 nm excitation light, the 1060 nm fluorescence signal enhanced with the increase of CO concentration, while the 1525 nm fluorescence signal under activation by 980 nm excitation light remained stable (serving as an internal standard), achieving specific response to CO (Fig. 5A) [45]. In the mouse model of acute liver injury, the probe successfully detected acetaminophen-induced CO by means of light-activated NIR-II fluorescence imaging, demonstrating the potential for light-controlled precise detection [46].

Xing et al. designed a multifunctional injectable hydrogel (TiN + Fe(CO)_5_ + ALG) integrating TiN NDs and Fe(CO)_5_ nanoparticles, which can simultaneously achieve anti-tumor and anti-drug-resistant bacteria treatment. Among them, TiN NDs are prepared by ultrasound-assisted liquid-phase exfoliation and have excellent photothermal conversion performance; Fe(CO)_5_ can release CO gas under the irradiation of NIR-II laser, exerting the effect of gas therapy (GT); the hydrogel matrix is sodium alginate (ALG), which can gel under the action of calcium ions in the TME, realizing local sustained release of therapeutic agents.​Tumors are closely related to bacterial infections. Bacterial infections often accelerate tumor progression, while in traditional treatment, anti-tumor and anti-bacterial therapies are usually carried out separately, and the advantages of combined therapy have gradually become prominent; at the same time, the emergence of drug-resistant bacteria also urgently requires new anti-bacterial materials. The design of this hydrogel just meets the above needs: the photothermal effect of TiN NDs can directly ablate tumor cells and bacteria, and the heat generated can also catalyze Fe(CO)_5_ to release CO, forming a “photothermal-gas” synergistic therapy to enhance the therapeutic effect; the injectability and self-healing ability of the hydrogel enable it to adapt to irregular tumor shapes and maintain long-term drug release *in vivo*. Experimental results showed that TiN + Fe(CO)_5_ + ALG hydrogel has good photothermal performance and CO release capacity, which not only effectively inhibits the growth of mouse breast tumors, but also shows excellent anti-bacterial effect on skin wounds and subcutaneous abscesses infected by methicillin-resistant *Staphylococcus aureus* (MRSA). After treatment, the tumor and infection areas of the mice almost completely healed, with no significant weight loss or other adverse reactions, fully confirming its good biosafety and therapeutic efficacy (Fig. 5B) [47].

However, it still has certain limitations in the regulation of therapeutic responsiveness, specifically, it is difficult to achieve precise regulation of on-demand therapy according to the release trend of overexpressed substances in the TME (such as H_2_O_2_, H_2_S). Therefore, developing CO delivery systems with TME responsiveness has become a key direction to break through this limitation [48].

Exogenous light stimulation (such as NIR-II lasers), due to its spatiotemporal controllability, has become an important means to trigger CO release and simultaneous imaging, showing significant advantages in precisely targeting treatment sites and controlling release timing. However, this mode has inherent limitations: the light penetration depth in deep tissues is restricted (for example, the penetration depth of 1064 nm laser in tumor tissue is usually less than 10 mm), and an external light source is required, making it difficult to apply in complex, deep-seated *in vivo* lesions (such as pancreatic tumors or solid organ infections). In contrast, endogenous microenvironment-responsive systems can rely on specific physiological features of diseased sites (such as high H_2_O_2_ and acidic pH in TME, and high H_2_S in infectious sites) to achieve autonomous and targeted CO release without external intervention, making them more suitable for complex *in vivo* lesion scenarios. The following will provide a detailed explanation of the design and application of endogenous microenvironment-responsive CO nanodelivery systems.

Exogenous light stimulation has become an important trigger for CO release due to its non-invasive nature and precise controllability; however, the issue of insufficient deep tissue penetration limits its application scenarios. In contrast, endogenous microenvironment-responsive mechanisms can achieve autonomous targeted release based on the unique physiological characteristics of the lesion site, without the need for external equipment, making it more suitable for complex *in vivo* lesions. The next section will provide a detailed explanation of the design and application of this response mode.

### 3.2 Endogenous microenvironmental responses

Endogenous microenvironment-responsive models use the unique physiological characteristics of diseased sites (such as high H_2_O_2_ and acidic pH in TME, and high H_2_S in infection sites) as triggering signals, enabling autonomous targeted CO release without external intervention, effectively addressing the issue of insufficient penetration of external light stimulation. This section will focus on the design logic of such responsive systems, analyzing how they achieve on-demand CO release through precise matching of ‘disease markers - release triggers,’ and discuss their application value in complex lesions.

In recent years, studies have found that there exists a special microenvironment in solid tumors, characterized by high-level production of hydrogen peroxide, excessive release of hydrogen sulfide gas, and formation of acidic conditions [49,50].

**Fig. 6:**
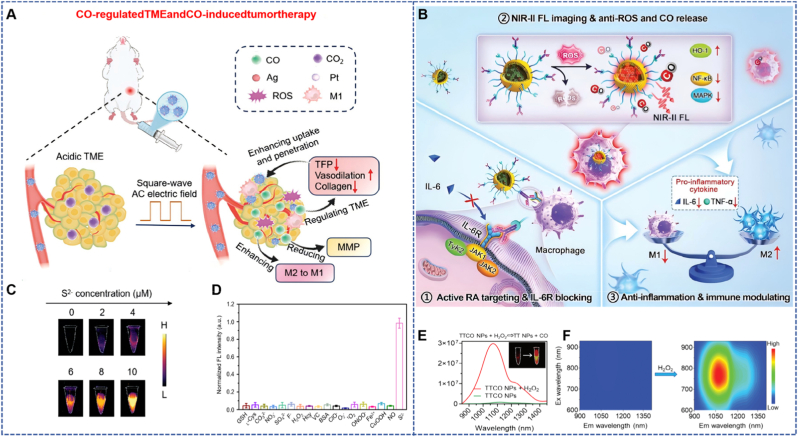
(A) AgPt@CaCO_3_-FA nanoparticles can release CO in response to the TME, which plays a synergistic therapeutic role, effectively regulates the TME and kills tumor cells [51]. Reprinted from with permission from Wiley. (B) TTCO NPs target rheumatoid arthritis sites for activation imaging and synergistic therapy in response to the microenvironment [52]. Reprinted from with permission from Wiley. (C) NIR-II FL images of AgPt@CaCO_3_-FA after treatment with different concentrations of Na_2_S under 808 nm laser excitation. (D) AgPt@CaCO_3_-FA nanoparticles have a highly selective response to S^2−^ in the TME. (E) TTCO NPs fluorescence response to H_2_O_2_ in rheumatoid arthritis enables NIR-II fluorescence imaging. (F) Responsiveness of TTCO NPs to H_2_O_2_ in the rheumatoid arthritis microenvironment.

Yang et al. designed a nanozyme based on AgPt@CaCO_3_ (AgPt@CaCO_3_-FA), which can be used for TME-responsive catalytic therapy, CO therapy, and IMT. The structural design of this nanozyme is quite targeted: AgPt alloy nanoparticles are loaded on CaCO_3_ nanospheres, and the surface-modified folic acid (FA) can significantly enhance its tumor targeting ability. In terms of functional performance, it is highly compatible with the characteristics of TME — under the acidic conditions of TME, AgPt@CaCO_3_-FA can react with endogenous H_2_S to generate Ag_2_S quantum dots, which can not only activate NIR-II fluorescence imaging but also catalyze the generation of CO gas from CO_2_ under the action of an electric field. Compared with the limitations of traditional nanozymes, such as low catalytic efficiency in tumor tissues and toxic side effects on normal tissues, this H_2_S-responsive NIR-II fluorescent nanozyme, through the specific release of CO at the tumor site, can not only regulate the TME and enhance its own catalytic efficiency but also achieve a synergistic effect between PTT and IMT (Fig. 6A) [51]. *In vitro* experiments showed that AgPt@CaCO_3_-FA exhibited significant catalase-like activity under the action of an electric field, which could efficiently generate ROS, thereby inducing oxidative stress damage and apoptosis of cells. *In vivo* experiments further confirmed that this nanozyme could significantly inhibit tumor growth, improve the survival rate of mice, and effectively suppress tumor metastasis; in addition, the released CO could regulate the TME by reprogramming tumor-supporting M2-type macrophages into killer M1-type macrophages, further enhancing the effect of IMT.

Tian et al. designed a dual-driven nanomotor (DSACGM NPs) based on diselenide MOF for multimodal tumor-targeted therapy. The nanomotor was constructed by a layer-by-layer assembly method, and its structure sequentially encapsulated NIR-II photothermal-responsive gold nanorods (AuRods), Mn_2_O_10_(MnCO), and glucose oxidase (GOD). The surface-modified 4T1 cancer cell membrane fragments endow it with tumor targeting ability. The dense extracellular matrix and high interstitial pressure of tumors can limit the penetration of nanoparticles, thereby reducing the therapeutic effect. Based on this, the design of this nanomotor aims to promote its own movement through the dual-driving effect of photothermal effect and CO release, enhancing the penetration ability into tumor tissues. At the same time, GOD can catalyze glucose oxidation, inducing tumor starvation while generating H_2_O_2_, which in turn can trigger CO release, enhancing the effect of low- temperature PTT by inhibiting the expression of HSPs; in addition, CO and Mn^2+^-triggered Fenton reaction can increase intracellular ROS levels, and diselenide further amplifies the effect of CDT by depleting glutathione (GSH), forming a multi-mechanism synergistic therapy system.​Experimental results showed that exhibited significant photothermal effect and CO release ability under NIR-II light irradiation; the GOD in the nanomotor maintained high activity, which could effectively consume glucose and generate H_2_O_2_. In 4T1 cells, DSACGM NPs significantly increased intracellular ROS levels, reduced heat shock protein 70 (HSP70) expression, and successfully induced cell apoptosis; in the 4T1 tumor mouse model, the nanomotor achieved significant accumulation at the tumor site, effectively ablated the tumor after NIR-II light irradiation, and the tumor volume was significantly reduced after treatment, with no significant change in the body weight of mice, confirming its good biocompatibility (Fig. 6B) [52].

In this type of CO nanodelivery system, the delivery level is limited to the tissue or cell level, which often causes associated damage to surrounding normal tissues. In addition, there are significant individual differences in the concentration of overexpressed substances, and their spatiotemporal distribution is uneven, which may lead to insufficient or excessive release of CO, thereby affecting the therapeutic effect or increasing the risk of toxic side effects. Therefore, developing delivery systems with organelle-targeted response ability has become an important breakthrough to improve treatment accuracy and reduce off-target effects [53].

Endogenous microenvironment-responsive systems achieve targeted release of CO at the tissue/cellular level through autonomous matching of “disease marker–release trigger,” effectively reducing toxic side effects on normal tissues. However, this approach still has limitations in precision: its response depends on microenvironmental differences at the tissue level (such as the difference in H_2_O_2_ concentration between tumors and normal tissues), and it cannot further distinguish organelle-specific needs within pathological cells—for example, mitochondria are key targets for CO to exert antitumor effects (such as disrupting the electron transport chain), while lysosome-targeted release can avoid non-specific damage to other organelles. Therefore, it is necessary to improve the response precision from the tissue/cellular level to the organelle level, achieving subcellular-level precise CO release through organelle-targeted design. The following text will elaborate on the design strategies and mechanisms of organelle-targeted precise responsive systems.

The endogenous microenvironment-responsive system achieves targeted CO release at the tissue/cellular level by autonomously recognizing lesion characteristics, significantly reducing damage to normal tissues. However, this model still struggles to differentiate the needs of different organelles within pathological cells and cannot achieve precise subcellular delivery. An organelle-targeted precision response model can precisely address this limitation, and the next section will delve into its design strategies and application effects.

### 3.3 Organelle targeting precision responses

To further improve the precision of CO delivery, organelle-targeted precise response systems refine the delivery level from the tissue/cell level to the subcellular level. By targeting key organelles such as mitochondria and lysosomes, CO is locally released at the core sites of pathology, enhancing therapeutic efficacy while minimizing damage to normal organelles. This section will introduce the design methods of such systems, the mechanisms of CO release, and application examples in the treatment of diseases such as tumors.

Inspired by the paradigm of enhancing targeting precision in the design of organelle-targeted probes, researchers have introduced the process of organelle-targeted response into existing CO nanosystems, accurately refining the delivery level to the organelle level, which improves the delivery accuracy of CO gas, enhances its therapeutic efficiency, and reduces the hazards of CO gas during delivery [[54], [55], [56]].

**Fig. 7:**
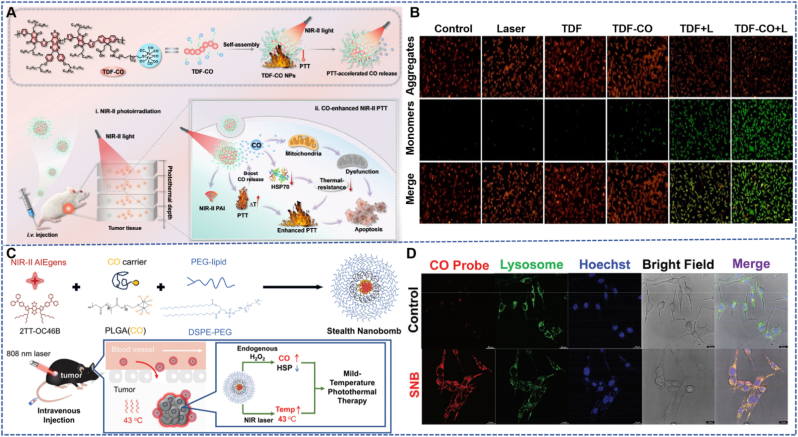
(A) TDF-CO NPs targeted tumors after injection through tail veins, releasing CO and generating heat in mitochondria under near-infrared irradiation, enhancing the PTT effect [57]. Reprinted from with permission from Springer. (B) Fluorescence image of 4T1 cells stained with JC-10 after co-culture with TDF-CO nanoparticles. (C) Assembly of “Stealth Nanobomb” and their mechanisms in response to organelle lysosome-triggered CO and photothermal synergistic therapy [40]. Reprinted from with permission from Wiley. (D) Distribution of SNB in lysosomes and the process of organelle-targeted precise responsive CO combination therapy.

The TDF-CO NPs developed by Zhu et al. comprise a semiconducting polymer framework, hydrophilic PEG chains, and a heat-responsive CO donor—its core advantages lie in specific mitochondrial targeting to 4T1 breast cancer cells and NIR-II light-triggered dual functions (CO release + PTT/PAI). Specifically, the thermosensitive CO donor in TDF-CO NPs can be triggered by 1064 nm NIR-II light to release CO; meanwhile, its NIR-II absorption (with a shoulder peak near 1064 nm) enables PTT with a photothermal conversion efficiency of 43.1 %, and PAI for real-time therapeutic guidance. Confocal imaging with JC-10 staining shows that TDF-CO NPs effectively induce mitochondrial dysfunction, with a significant decrease in mitochondrial membrane potential, confirming their excellent mitochondrial targeting. Taking advantage of the high ROS microenvironment in mitochondria of 4T1 cells, TDF-CO NPs accumulate in mitochondria after endocytosis, and NIR-II light irradiation triggers CO release: CO acts on mitochondria to disrupt the electron transport chain, promoting intracellular ROS generation and inducing apoptosis; simultaneously, CO inhibits the overexpression of HSP70 (downregulated compared to the TDF + laser group), reducing cellular thermotolerance and enhancing PTT efficacy, thereby avoiding non-specific damage to other organelles. In addition, the amphiphilic structure of TDF-CO NPs ensures stable circulation in the bloodstream, preventing premature CO release (Fig. 7A). *In vitro* 4T1 cell experiments showed that after TDF-CO NPs released CO in mitochondria under NIR-II irradiation, the intracellular ROS level significantly increased, the early apoptosis rate reached 79.3 %, and the cell viability was significantly reduced with an IC50 (half-maximal inhibitory concentration) of 43.45 μg/mL; in 4T1 tumor-bearing mice, the mitochondrial CO concentration in tumor tissues was sufficient to enhance PTT, leading to complete tumor eradication in some mice after treatment, while H&E staining showed no obvious damage to mitochondria in normal organs [57].

The SNB developed by Ma et al. (loaded with PLGA (CO) and NIR-II AIEgens) exhibits two key features for lysosome-targeted CO delivery to pancreatic cancer cells: high lysosomal targeting specificity and H_2_O_2_-responsive CO release coupled with NIR-II imaging/PTT. First, its lysosomal targeting is verified by a co-localization coefficient of 0.916 with lysosomal markers (via confocal imaging); second, the Fe-CO coordination structure in PLGA (CO) undergoes a Fenton-like reaction in the high H_2_O_2_ (10 μM) lysosomal environment to release CO, while AIEgens generate 960 nm NIR-II fluorescence for imaging and achieve a photothermal conversion efficiency of 33.1 % for PTT. Taking advantage of the high H_2_O_2_ microenvironment in lysosomes of pancreatic cancer cells, SNB accumulates in lysosomes after endocytosis, and H_2_O_2_ triggers CO release: CO diffuses from lysosomes to the cytoplasm, weakening cellular thermal tolerance by inhibiting HSP70 expression (downregulated by 60 %); at the same time, the acidic environment of lysosomes (pH 4.5–5.0) can enhance the hydrolysis of PLGA (CO), accelerating CO release, thereby avoiding non-specific effects on other organelles. In addition, the PEG shell can prevent nanoparticles from being degraded by lysosomes prematurely, ensuring controlled release of CO in lysosomes (Fig. 7C). *In vitro* Panc02 cell experiments showed that after SNB released CO in lysosomes, the expression of HSP70 was only 0.4 times that of the Control group, the cell death rate reached 85 % under 43 °C moderate temperature PTT conditions, and the lysosomal membrane permeability increased by 3.2 times (verified by Acridine Orange (AO) staining); in mice with *in vivo* pancreatic cancer models, the CO concentration in lysosomes of tumor tissues reached 4.1 μM, the tumor volume shrank to 1/5 of the Control group after 14 days of treatment, H&E staining showed that the lysosomal structure of tumors was destroyed, while lysosomes in normal tissues showed no abnormalities.

The three types of response patterns each have their focus: NIR-II has the best spatiotemporal controllability under light stimulation, but its tissue penetration depth (<10 mm) is limited; responses to endogenous microenvironments can adapt to complex lesions, but are affected by individual differences in microenvironmental marker concentrations; organelle targeting offers the highest precision, yet faces fluctuations in targeting efficiency due to cellular heterogeneity. NIR-II imaging's irreplaceability lies in its superior resolution in deep tissues compared to Magnetic Resonance Imaging (MRI), real-time dynamic monitoring capabilities beyond Positron Emission Tomography (PET), and its ability to integrate with phototherapy modules to achieve a ‘imaging-treatment-feedback’ closed loop, although its sensitivity for detecting low concentrations of CO still needs improvement.

This chapter revolves around the core objective of ‘on-demand precise CO release,’ systematically explaining the design logic and application scenarios of three mainstream response modes: Exogenous light stimulation (primarily using NIR-II lasers) leverages spatiotemporal controllability to achieve synchronized control of CO release and imaging, suitable for superficial lesions or deep lesions accessible with endoscopic assistance, but limited by tissue penetration depth; Endogenous microenvironment-responsive systems rely on the unique physiological characteristics of lesion sites (such as high H_2_O_2_ levels and acidic pH in the tumor microenvironment) to achieve autonomous targeted CO release without the need for external equipment, making them more suitable for deep and complex lesions; Organelle-targeted precise responses elevate delivery accuracy from the tissue/cell level to the subcellular level (such as mitochondria-targeted TDF-CO nanoparticles and lysosome-targeted SNB), enhancing CO therapeutic efficacy through localized release while minimizing damage to normal organelles. Each of these three response modes has its focus and complements the others, together forming the core technological support for ‘precise drug release’ in CO nanodelivery systems. The next section will focus on how to leverage these efficient and controllable response mechanisms to integrate CO gas therapy with modalities such as PTT, PDT, CDT, and IMT, further improving therapeutic outcomes while reducing systemic toxicity.

## 4 Synergistic research on CO gas therapy and multimodal therapies under the NIR-II window

With the optimization of CO nanocarrier designs and the realization of precise CO release via multiple response modes, combining CO gas therapy with other therapeutic modalities under NIR-II guidance has become a key strategy to enhance efficacy and reduce systemic toxicity—this integration not only compensates for the limitations of single therapies (e.g., PTT thermal tolerance, PDT hypoxia sensitivity), but also leverages NIR-II imaging to achieve real-time monitoring of synergistic therapeutic processes. This section focuses on the mechanisms and applications of synergistic therapies between CO and PDT/PTT/CDT/IMT.

### 4.1 CO combined with PDT, PTT

Based on NIR-II imaging-guided and precise CO release mechanisms, combining CO gas therapy with PDT and PTT has become a key strategy to overcome the limitations of single therapies. PDT is easily restricted by the hypoxic tumor environment, and PTT often leads to thermal resistance due to HSPs upregulation. CO, on the other hand, can improve the hypoxic microenvironment, extend the action time of ROS, and inhibit HSPs expression, thereby enhancing cellular sensitivity to heat. This section will detail the synergistic design logic of CO with PDT and PTT, representative nanoplatform construction, and their therapeutic effects in cancer treatment.

After clarifying the precise response mechanism of organelle targeting, the application value of CO in multimodal combined therapy has been further highlighted [[58], [59], [60]]. The following elaborates on the research progress regarding the combined application of CO with PDT, PTT, CDT, and immunotherapy.

**Fig. 8:**
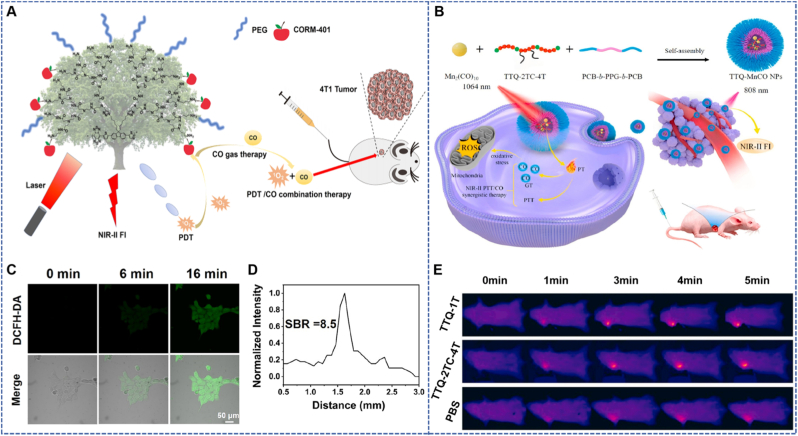
(A) Under light conditions, ROS produced by PBF-AM3 can initiate PDT and trigger the release of CO from CORM-401 for gas therapy, which synergistically significantly inhibits 4T1 tumor growth [61]. Reprinted from with permission from ACS Publications. (B) TTQ-MnCO NPs achieve synergistic anti-tumor resistance with PTT by releasing CO and thermogenesis under near-infrared light [64]. Reprinted from with permission from Elsevier. (C) Under light conditions, PBF-g-CO/PEG can induce intracellular ROS production, and with time, the ROS level gradually increased. (D) Quantitative curves of NIR-II fluorescence intensity of PBF-g-CO/PEG injected into the tail vein in 4T1 tumor mice at different time intervals. (E) TTQ-1T NPs and TTQ-2TC-4T NPs showed different photothermal effects over time in solution under NIR irradiation, while PBS showed no significant change.

Du et al. designed a NIR-II fluorescent conjugated polymer brush (PBF-g-CO/PEG) modified with polyamidoamine (PAMAM) dendrimers, which is applied for the synergy between PDT and CO gas therapy. The polymer covalently links the CO donor (CORM-401) and polyethylene glycol (PEG) to the NIR-II fluorescent polymer brush (PBF-AM3), enabling NIR-II fluorescence imaging (NIR-II FI)-guided combined PDT/CO gas therapy. To address the issues that ROS generated by PDT have a short lifespan and the hypoxia in solid tumors limits the therapeutic efficacy, the combination with CO gas therapy not only ameliorates the hypoxic state but also achieves the synergistic release of ROS and CO through light triggering, thereby significantly enhancing the therapeutic effect. Experiments demonstrated that PBF-g-CO/PEG can efficiently generate ROS under 660 nm light irradiation, while triggering CORM-401 to release CO, which induces cancer cell apoptosis; the inhibitory effect on 4T1 cell viability under light conditions is significantly superior to that of PDT or CO therapy alone, and apoptosis experiments further confirmed its high cytotoxicity upon light exposure (Fig. 8A) [61]. In the 4T1 tumor mouse model, after tail vein injection, PBF-g-CO/PEG can accumulate at the tumor site, and under 660 nm light irradiation, it realizes NIR-II fluorescence imaging-guided combined PDT/CO gas therapy, which significantly inhibits tumor growth with favorable biocompatibility [62,63].

Sun et al. developed conjugated polymer nanoparticles (TTQ-1T NPs and TTQ-2TC-4T NPs) by encapsulating TTQ-1T and TTQ-2TC-4T in amphiphilic block copolymer PCB-b-PPG-b-PCB respectively. These nanoparticles exhibit photothermal effects under 1064 nm laser irradiation for PTT. *In vitro*, TTQ-1T NPs (0.06 mg/mL) and TTQ-2TC-4T NPs (0.06 mg/mL) show temperature elevations to 43.4 °C and 35.8 °C, respectively, after 5 min of 1064 nm laser irradiation (1 W/cm^2^). *In vivo*, for MCF-7 tumor-bearing mice, 24 h after intravenous injection, the tumor site temperatures reach 62.8 °C and 56.8 °C within 5 min under 1064 nm laser irradiation, resulting in tumor inhibition rates of 90.52 % and 82.84 %, respectively. Furthermore, they constructed by co-encapsulating TTQ-2TC-4T and heat-responsive CO donor (Mn_2_(CO)_10_) into PCB-b-PPG-b-PCB, achieving 1064 nm laser-triggered NIR-II PTT/Gas synergistic therapy. TTQ-MnCO NPs (0.06 mg/mL) exhibit a temperature increase of 35.5 °C under 1064 nm laser irradiation (1 W/cm^2^) for 5 min, with a high photothermal conversion efficiency of 44.43 % and good stability over five heating-cooling cycles. *In vivo*, 24 h after injection of TTQ-MnCO NPs into MCF-7 tumor-bearing mice, the tumor temperature rises to 56 °C under 1064 nm laser irradiation for 5 min, leading to a high tumor inhibition rate of 94.83 % due to the synergistic effect of NIR-II PTT and CO release (Fig. 8B) [64,65].

The synergy between CO and PDT/PTT essentially achieves a therapeutic gain of ‘1 + 1>2’ through functional complementation - CO overcomes the core bottlenecks of traditional phototherapy, while NIR-II imaging provides real-time monitoring for the synergistic process. Beyond phototherapy, CO's characteristics in modulating the tumor microenvironment and inducing immunogenic cell death also lay the foundation for its combination with CDT and IMT. The next section will focus on the mechanisms and practical applications of these two types of synergistic models.

### 4.2 CO combined with CDT and IMT

CDT relies on ROS in the tumor microenvironment to achieve targeted therapy but is often limited by insufficient ROS production; IMT faces the obstacle of an immunosuppressive tumor microenvironment. CO gas therapy can precisely address these shortcomings: on one hand, it can amplify CDT-induced oxidative stress damage by modulating the microenvironment and consuming antioxidants; on the other hand, it can induce immunogenic cell death and remodel the immune microenvironment to enhance IMT. This section will explore the synergistic mechanisms of CO with CDT and IMT, as well as the design and application of related nanoplatforms.

**Fig. 9:**
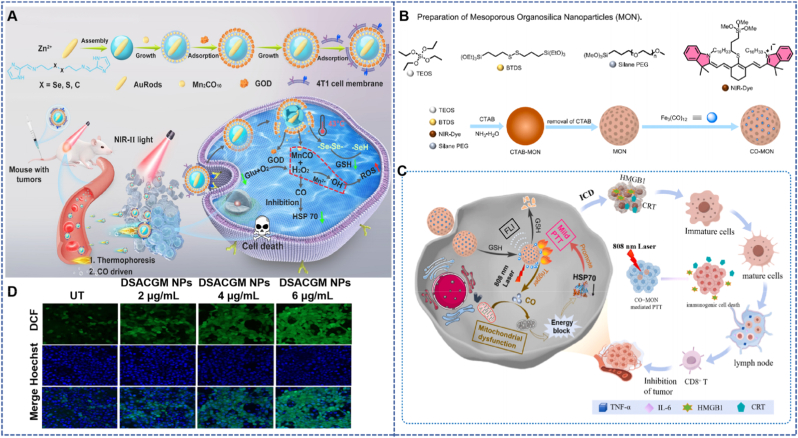
(A) DSACGM NPs penetrate tumor tissue driven by NIR-II and CO, facilitating multimodal synergistic therapy including starvation therapy, CDT and PTT [66]. Reprinted from with permission from Science China Press. (B) CO-MON synthesis schematic diagram. (C) CO-MON-based PTT induced tumor immunogenic cell death [67]. Reprinted from with permission from Elsevier. (D) Representative images of DCF fluorescence (ROS) measured at different concentrations of DSACGM NPs.

The dense extracellular matrix and elevated interstitial pressure in the TME severely hinder nanoparticle penetration, limiting therapeutic efficacy. To address this, Tian et al. [66]. developed a diselenide MOF-based dual-driven nanomotor (DSACGM NPs) *via* layer-by-layer assembly, with a focus on CDT synergism. This system comprises a Zn^2+^-coordinated diselenide MOF scaffold encapsulating NIR-II-responsive gold nanorods, MnCO, and GOD, surface-functionalized with 4T1 cell membranes for tumor targeting. Central to its mechanism is the amplification of CDT: GOD catalyzes glucose oxidation to generate H_2_O_2_, which triggers MnCO decomposition, releasing Mn^2+^ and CO. Mn^2+^ mediates Fenton-like reactions in the acidic tumor microenvironment, converting H_2_O_2_ into highly toxic ·OH. Concurrently, diselenide components deplete intracellular GSH, a key antioxidant, thereby exacerbating oxidative stress and potentiating CDT efficacy. The dual driving force from NIR-II-induced photothermal effects and CO release enhances tumor penetration, facilitating CDT agent delivery (Fig. 9A) .

*In vitro* studies confirmed that DSACGM NPs significantly elevated intracellular ROS levels in 4T1 cells under NIR-II irradiation, inducing apoptosis. *In vivo*, the nanomotor achieved targeted accumulation, efficiently ablating tumors at sub-hyperthermic temperatures (<45 °C) through enhanced CDT, with negligible systemic toxicity. This design highlights the potential of integrating gas release and GSH depletion to amplify CDT, offering a promising strategy for overcoming tumor microenvironment barriers [67].

The study by Gan et al. revealed the synergistic role of CO in immunogenic cell death (ICD): CO released by the CO-MON nano-platform under 808 nm laser irradiation can inhibit the upregulation of HSP70 in tumor cells, enhance the damage of mild PTT to tumor cells, and thus efficiently induce ICD. Its immune effect is significant: after ICD occurs, tumor cells release damage-associated molecular patterns (DAMPs), including exposed calreticulin (CRT) and secreted high mobility group box 1 (HMGB1), among which the green fluorescent signal of CRT is significantly enhanced, and the level of HMGB1 reaches 29 pg/mL (1.5 times that of other groups). These DAMPs activate the maturation of dendritic cells (DCs), so that the pro-inflammatory factors TNF-α, 624 pg/mL and IL-6, 593 pg/mL secreted by DCs are increased by 1.5 times and 1.15 times respectively, the level of interleukin-10 (IL-10) is decreased by 1.2 times, and the proportion of CD80/CD86 double-positive mature DCs is 2–3 times that of other groups. Mature DCs further recruit cytotoxic CD8^+^ T lymphocytes, increasing the infiltration of CD8^+^ T cells in the TME by 7.21 % (about twice that of the control group), effectively activating the adaptive anti-tumor immune response and enhancing the ability of tumor immune clearance (Fig. 9B) [67].

This section focuses on the synergistic mechanisms and applications of CO combined with multimodal therapy guided by NIR-II: when CO is combined with PDT, it can overcome the limitations of traditional PDT efficacy by improving the tumor hypoxic environment and prolonging the effect of ROS; when combined with PTT, CO can inhibit the expression of heat shock proteins and reduce tumor cell thermotolerance, achieving enhanced low-temperature photothermal effects; when combined with CDT, CO amplifies oxidative stress damage through microenvironment regulation and GSH depletion; meanwhile, CO's regulation of ICD provides possibilities for its combination with IMT. These synergistic strategies not only enhance the therapeutic efficacy of single treatments but also offer a new approach for precision therapy by “reducing dosage and toxicity”. [68,69].

The synergistic effects of CO with different modalities vary: its core advantage in combination with PTT lies in inhibiting HSPs expression and reducing heat resistance, a mechanism that has been well validated; synergy with CDT depends on GSH depletion to amplify oxidative damage, but its effectiveness can be limited in hypoxic tumor microenvironments due to insufficient H_2_O_2_ production; synergy with immunotherapy works by inducing ICD to activate antitumor immunity, although its effect on tumors resistant to immune checkpoint inhibitors remains controversial.

The synergistic integration of CO with CDT and IMT further expands the boundaries of NIR-II guided precision therapy — CDT utilizes CO to amplify oxidative damage, while IMT relies on CO to break immune tolerance. The combination of both with CO not only strengthens local treatment effects but also activates systemic anti-tumor immunity. In summary, the synergistic strategy of CO and multimodal therapies, through multiple functions of ‘microenvironment modulation, targeted damage, and immune activation,’ provides new ideas for the precise treatment of complex diseases and lays a foundation for summarizing future research challenges and directions.

## 5 Conclusion

NIR-II imaging-guided CO nanodelivery systems have emerged as a powerful platform in precision nanomedicine, combining high-resolution deep-tissue imaging with the therapeutic potential of CO for the treatment of cancer, infections, and inflammation-related diseases. This review systematically summarizes the structural design strategies of these systems, including polymers, core-shell structures, MOFs, and peptide-modified nanoplatforms, each offering unique advantages in enhancing biocompatibility, loading capacity, and targeted delivery efficiency.

In terms of delivery responsiveness, these systems have demonstrated effective control of CO release through exogenous light activation, endogenous microenvironment responsiveness, and organelle-targeted precision mechanisms, enabling on-demand, site-specific CO delivery. Notably, organelle-targeted delivery, such as mitochondrial and lysosomal targeting, has significantly improved delivery precision while minimizing off-target effects and toxicity.

The synergistic integration of CO therapy with PTT, PDT, CDT, and IMT under NIR-II guidance has been shown to enhance therapeutic efficacy while reducing the necessary treatment dosages, thereby minimizing systemic side effects. CO's unique ability to modulate the TME, inhibit heat shock proteins, regulate immune cell polarization, and disrupt biofilms complements these modalities, facilitating more effective and comprehensive treatment strategies.

Polymer-based vectors have the highest clinical translation potential due to FDA approval background, mature large-scale synthesis processes, and clear biocompatibility data, but their risks are mainly focused on lot-to-lot differences in CO release kinetics and possible long-term accumulation in liver and spleen tissue. Although MOF-based carriers have excellent loading capacity and stimulus response, the biocompatibility of metal nodes has not been fully verified, and the *in vivo* degradation pathway lacks standardized studies, which has potential systemic toxicity risks and a higher threshold for clinical translation. The core-shell structure and peptide modification system improves the precision of treatment through functional integration and targeted optimization, but it is difficult to meet the needs of large-scale clinical applications due to the low yield and high cost caused by complex preparation processes. In terms of imaging advantages, the NIR-II imaging system has irreplaceable value compared with NIR-I and traditional modalities such as MRI and PET: compared with NIR-I, NIR-II light has a 5–10 times higher tissue penetration depth and significantly reduced photon scattering, which can achieve accurate imaging of deep lesions that cannot be covered by NIR-I. Compared with MRI, the deep tissue imaging resolution is better and there is no radiation damage. Compared with PET, it can achieve real-time dynamic monitoring within 5 min, which is more suitable for the closed-loop treatment of CO “on-demand release”, which is difficult to achieve with traditional imaging technology.

Despite these significant advancements, challenges remain, including the heterogeneity of disease microenvironments affecting delivery efficiency, potential risks of uncontrolled CO release, and the complexity of platform fabrication processes. Moreover, two critical bottlenecks need targeted solutions: (1) The lack of real-time subcellular CO monitoring—current NIR-II imaging can only track nanoplatform distribution, but cannot directly quantify CO concentrations in organelles, making it impossible to adjust the therapeutic strategy in real time based on actual CO release at the target site; (2) Unfinished standardization for clinical translation—key parameters such as nanoplatform degradation rate *in vivo*, maximum tolerated CO dose in humans, and NIR-II imaging-guided treatment protocols (laser power, irradiation time) have not yet been standardized, leading to difficulties in multi-center clinical trial validation.

In summary, NIR-II imaging-guided CO nanodelivery systems have demonstrated outstanding potential in enhancing the precision and efficacy of gas therapy, providing innovative solutions for the treatment of challenging diseases while reducing adverse effects. The continuous evolution of nanoplatform design and responsiveness, coupled with the synergistic integration of imaging and therapy, underscores the pivotal role of these systems in advancing the field of precision medicine and expanding the therapeutic applications of gas therapy in biomedical research.

## 6 Outlook

**Scheme 3:**
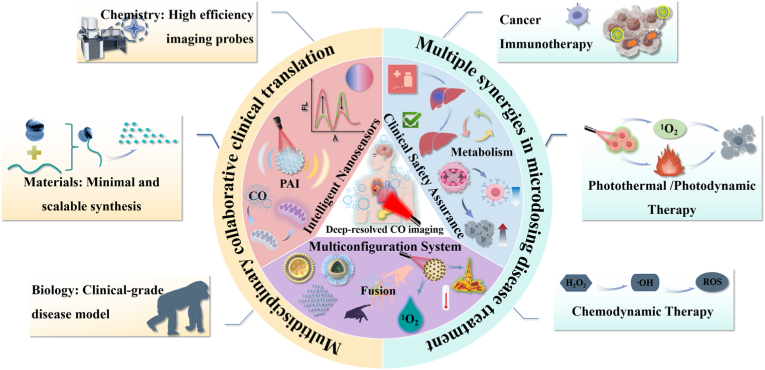
The future development trend of carbon monoxide nanodelivery systems combined with NIR - II imaging.

The integration of NIR-II imaging with CO nanodelivery systems marks a significant advancement in the pursuit of precise, image-guided gas therapy. The fundamental advantage of this system—distinguishing it from traditional gas therapy and other light-controlled platforms—lies in its ability to balance “therapeutic efficacy” and “precision safety”: unlike traditional gas therapy that “blindly delivers” CO, NIR-II guidance achieves subcellular-level release regulation, minimizing off-target toxicity; compared with NIR-I/UV light-controlled systems limited by shallow penetration, NIR-II's deep tissue accessibility allows application to more complex disease scenarios, expanding the clinical application boundary of gas therapy. This unique “deep penetration + visualized control” feature is the core driving force for its translation from preclinical research to clinical practice. However, translating these technologies from bench to bedside requires addressing several concrete bottlenecks and actionable directions, while expanding biomedical application scenarios to accelerate clinical translation: (1)Targeting Core Technological Bottlenecks with Actionable Research Directions:Firstly, lack of real-time subcellular CO monitoringCurrent NIR-II imaging only tracks nanoplatform distribution but cannot directly quantify CO concentrations in specific organelles (e.g., mitochondria vs. cytoplasm) or capture dynamic release kinetics (e.g., how CO release rates correlate with NIR-II laser intensity or microenvironmental H_2_O_2_ levels). This makes it impossible to define the “effective therapeutic window” for CO or adjust treatment strategies in real time. Actionable direction: Develop intelligent NIR-II responsive nanosensors integrating ratiometric fluorescence/photoacoustic signals with CO-sensitive probes (e.g., Pd^2+^-coordinated ligands or CO-responsive AIEgens). These sensors should enable real-time visualization of CO concentration gradients in mitochondria and lysosomes of tumor/infected tissues, laying the foundation for closed-loop feedback-controlled delivery systems. Secondly, insufficient synergy between NIR-II imaging and CO bioactivityExisting platforms often treat imaging and therapy as independent modules—NIR-II signals only indicate nanoplatform accumulation, not whether CO has been released or exerted biological effects (e.g., inhibiting HSPs or inducing ICD). Actionable direction: Design “theranostic-coupled” nanoplatforms where CO release directly modulates NIR-II signals. For example, CO-triggered cleavage of coordination bonds between metal nodes and fluorophores can alter NIR-II emission wavelengths, enabling real-time confirmation of CO bioavailability while imaging. On the other hand, unstandardized clinical translation parametersKey parameters such as *in vivo* nanoplatform degradation rate, maximum tolerated CO dose in humans, and NIR-II imaging-guided treatment protocols (laser power, irradiation time, administration route) lack unified standards, hindering multi-center clinical trial validation. Actionable direction: Conduct systematic preclinical studies using patient-derived xenograft (PDX) models and orthotopic tumor models to establish dose-response relationships (e.g., CO concentration vs. therapeutic effect/toxicity) and standardize imaging-therapy parameters. Collaborate with clinical engineers to optimize NIR-II imaging devices for clinical use (e.g., miniaturized endoscopic NIR-II systems for deep-seated tumors). At the same time, it is necessary to establish a ‘nanoplatform–disease type’ matching evaluation system — for example, for superficial tumors, priority should be given to developing NIR-II light-responsive polymer carriers, highlighting their ease of preparation and low toxicity; for deep-seated solid tumors, the focus should be on optimizing the degradation rate of metal nodes and targeting efficiency of MOF-based carriers; for infectious diseases, the emphasis should be on developing inhalable or locally injectable CO-releasing systems to reduce systemic exposure risk. This categorized evaluation approach can provide a more precise basis for technology selection in clinical translation. (2)Expanding Biomedical Application Scenarios for Clinical Translation:Firstly, chronic Lung Diseases Post-COVID-19Post-COVID pulmonary complications (e.g., interstitial fibrosis, microvascular remodeling) and chronic lung disorders (IPF, ALI, COPD) share pathological features of oxidative stress, immune dysregulation, and persistent inflammation—targets perfectly matched to CO's anti-inflammatory, anti-fibrotic, and immunomodulatory properties. Translation focus: Develop inhalable NIR-II-responsive CO nanocarriers (e.g., lipid nanoparticles or hydrogel microspheres) for non-invasive delivery to the lungs. NIR-II imaging can monitor lung distribution and CO release, while localized CO delivery minimizes systemic exposure. Clinical trials should prioritize patients with mild-to-moderate post-COVID fibrosis to evaluate safety and efficacy in reducing lung inflammation and fibrosis progression. Secondly, orthopedic Implant-Related InfectionsTraditional orthopedic materials (titanium alloys, PMMA bone cement) are prone to postoperative infections and stress shielding. CO nanocarriers (e.g., MOF-coated 3D-printed implants) can precisely release CO to inhibit biofilm formation and regulate inflammation, while NIR-II imaging monitors infection resolution. Translation focus: Collaborate with orthopedic device manufacturers to develop CO-releasing composite implants. Conduct preclinical studies using animal models of implant-associated MRSA infections to validate infection control, bone integration, and long-term biocompatibility. Aim for early-phase clinical trials in patients undergoing joint replacement or fracture fixation at high risk of infection. Last but not least, refractory Inflammatory DiseasesRheumatoid arthritis (RA) and inflammatory bowel disease (IBD) involve localized overexpression of pro-inflammatory factors (TNF-α, IL-6) and oxidative stress. CO's ability to downregulate these factors and modulate macrophage polarization (M2→M1) makes it a promising therapeutic agent. Translation focus: For RA, develop intra-articular injectable NIR-II-responsive hydrogels loaded with CO prodrugs, enabling targeted release triggered by the acidic joint microenvironment. For IBD, design oral colon-targeted CO nanocarriers (e.g., pH-sensitive polymers) combined with NIR-II endoscopic imaging to guide local treatment. Clinical translation should start with small-scale trials in patients unresponsive to conventional anti-inflammatory drugs. (3)Strengthening Cross-Disciplinary Collaboration for Translation:The successful translation of NIR-II-guided CO nanoplatforms requires synergy across materials science, chemistry, biomedical engineering, and clinical medicine; Materials scientists should focus on biodegradable and scalable nanoplatform synthesis (e.g., PLGA-MOF composites approved by the FDA for faster clinical entry); Clinical researchers should define unmet medical needs (e.g., refractory infections, post-COVID complications) and design clinical trial protocols aligned with regulatory requirements; Engineers should optimize NIR-II imaging devices for bedside use, such as portable NIR-II imagers for real-time monitoring during treatment; Collaborate with the clinical laboratory team to establish efficacy evaluation standards for CO therapy, integrating indicators such as ‘subcellular CO concentration monitoring data’, ‘inflammatory factor change curves’, and ‘lesion tissue pathology scores’ into a quantitative assessment system to address the current lack of unified standards for efficacy judgment. At the same time, promote early communication with regulatory authorities to clarify the clinical application pathway for CO nanomedicine, key points for toxicological evaluation, and biosafety inspection requirements, thereby shortening the translation cycle;

In conclusion, addressing core technological bottlenecks with actionable research directions and expanding application scenarios to clinically relevant diseases will accelerate the translation of NIR-II-guided CO nanomedicine. By integrating “precision monitoring + targeted therapy + clinical adaptability”, these systems hold great promise to revolutionize personalized treatment for cancer, infections, and inflammation-related diseases, ultimately transforming the landscape of nanomedicine (Scheme 3).

In conclusion, by addressing these multifaceted challenges, NIR-II imaging-guided CO nanodelivery systems hold the potential to revolutionize personalized therapy for cancer, infections, and inflammation-related diseases. Continued innovation in nanomaterial engineering, responsive system design, and imaging technology integration will pave the way for the realization of precise, effective, and safe gas therapy strategies in clinical settings, ultimately transforming the landscape of nanomedicine.

## CRediT authorship contribution statement

**Yaoqiang Li:** Writing – original draft. **Yeneng Dai:** Writing – original draft. **Bo Wang:** Writing – original draft. **Nan Zhang:** Writing – original draft. **Kangyi Yan:** Writing – original draft. **Haoqin Chen:** Writing – original draft. **Hongrong Shi:** Writing – original draft. **Hongli Chen:** Writing – original draft. **Qingzhi Wang:** Writing – original draft. **Jierui Yan:** Writing – original draft. **Xiaobo Wang:** Writing – original draft. **Peiyang Gao:** Writing – original draft. **Gongcheng Ma:** Writing – review & editing. **Ya Hou:** Writing – review & editing. **Qihang Ding:** Writing – review & editing, Supervision, Project administration, Funding acquisition. **Qi Zhao:** Writing – review & editing, Project administration, Funding acquisition.

## Declaration of competing interest

The authors declare that they have no known competing financial interests or personal relationships that could have appeared to influence the work reported in this paper.

## Data Availability

Data will be made available on request.
